# Analytical Investigation of the Profile of Human Chorionic Gonadotropin in Highly Purified Human Menopausal Gonadotrophin Preparations

**DOI:** 10.3390/ijms25179405

**Published:** 2024-08-29

**Authors:** Angela Capolupo, Sofia Petrocchi, Maura Melchiorre, Kim Jonas, Thomas D’Hooghe, Aylin Hanyaloglu, Sesh Sunkara, Angelo Palmese, Beste Ozgumus, Angela Amoresano, Gabriella Angiuoni, Susana Montenegro, Patrizia Simone, Monica Lispi

**Affiliations:** 1 Characterization & Innovative Analytics Unit—Analytical Development Biotech—Global Analytical Development—Global Development & Launch—Global Healthcare Operation, Merck Serono S.p.A., 00012 Guidonia, Italy, an affiliate of Merck KGaA, Darmstadt, Germany; angela.capolupo@merckgroup.com (A.C.); sofia.petrocchi@merckgroup.com (S.P.); maura.melchiorre@merckgroup.com (M.M.); gabriella.angiuoni@merckgroup.com (G.A.); patrizia.simone@merckgroup.com (P.S.); 2 Department of Women and Children’s Health, School of Life Course and Population Health Sciences, King’s College London, London WC2R 2LS, UK; 3 Global Medical Affairs—Fertility, Merck KGaA, 64293 Darmstadt, Germanysusana.montenegro@merckgroup.com (S.M.); 4 Department of Development and Regeneration, Laboratory of Endometrium, Endometriosis & Reproductive Medicine, KU Leuven, 3000 Leuven, Belgium; 5 Department of Obstetrics, Gynecology, and Reproductive Sciences, Yale University Medical School, New Haven, CT 06520-8065, USA; 6 Department of Metabolism, Digestion and Reproduction, Imperial College, London SW7 2AZ, UK; a.hanyaloglu@imperial.ac.uk; 7 Department of Chemical Sciences, University of Naples Federico II, Via Cintia, 80126 Naples, Italy; beste.ozgumusnitride@unina.it (B.O.); angela.amoresano@unina.it (A.A.); 8 Unit of Endocrinology, International PhD School in Clinical and Experimental Medicine, University of Modena and Reggio Emilia, 41121 Modena, Italy

**Keywords:** highly purified human menopausal gonadotropin, luteinizing hormone, placental human chorionic gonadotropin, glycosylation profiling

## Abstract

Highly purified human menopausal gonadotropin (HP-hMG [Menopur^®^, Ferring Pharmaceuticals, Saint-Prex, Switzerland]) contains a 1:1 ratio of follicle-stimulating hormone (FSH) and luteinizing hormone (LH). This analysis aimed to assess gonadotropin (FSH, LH and hCG) abundance in HP-hMG and clarify the source of hCG by assessing the presence of sulfated glycans, which are diagnostic for pituitary hCG forms due to their distinct glycosylation patterns. Additionally, the purity of each sample, their specific components, and their oxidation levels were assessed. HP-hMG samples (three of Menopur^®^ and two of Menogon^®^ Ferring Pharmaceuticals, Saint-Prex, Switzerland) were included in the current analyses. Brevactid^®^ (urinary hCG; Ferring Pharmaceuticals, Saint-Prex, Switzerland) and Ovidrel^®^ (recombinant hCG; Merck KGaA, Darmstadt, Germany) were used as control samples. Glycopeptide mapping and analysis of impurities were carried out by liquid chromatography–tandem mass spectrometry (LC-MS/MS). Oxidation was assessed through reducing peptide mapping using LC-MS/MS. The FSH and LH in the HP-hMG samples showed sulfated glycans, while no signals of sulfated glycopeptides were detected on any site of the beta subunit of hCG. HP-hMG test samples presented the same hCG glycan distribution as the control sample (placental hCG, Brevactid^®^) extracted from the urine of pregnant women, suggesting a non-pituitary source of hCG. Protein impurities were estimated to constitute approximately 20–30% of the entire HP-hMG protein content in the test samples. More than 200 non-gonadotropin proteins were identified in the HP-hMG test samples, of which several were involved in embryonic development or pregnancy. The alpha subunit of the tested samples was strongly oxidized, with a relative abundance of 20% of the total gonadotropin content. Without taking into account all the protein impurities, the beta subunit of LH was detected only in traces (0.9–1.2%) in all tested HP-HMG samples, confirming the data obtained by intact molecule analysis, while high levels of beta hCG (18–47%) were observed. Advanced molecular analysis of HP-hMG indicates a primarily placental origin of hCG, as evidenced by the absence of hCG sulfated glycans and the predominance of placental non-sulfated hCG in LH activity. The analysis revealed 20–30% of protein impurities and a significant presence of oxidized forms in the HP-hMG samples. These findings are critical for understanding the quality, safety, and clinical profile of HP-hMG.

## 1. Introduction

Follicle-stimulating hormone (FSH), along with luteinizing hormone (LH) and human chorionic gonadotropin (hCG), play a central role in mammalian reproduction. Exogenous FSH has been used to treat infertility in women since the 1960s. The first preparations available for clinical use were extracted from the pituitary glands of animals and later from the urine of postmenopausal women (i.e., “human menopausal gonadotropin” [hMG]) [1]. Initially, the preparations of hMG available for clinical use exhibited low levels of purity, containing numerous non-related gonadotropin contaminants and oxidized FSH forms [1,2]. Recombinant and highly purified urinary products were introduced for ovarian stimulation to address safety concerns associated with hMG [1]. Recombinant gonadotropins were produced through recombinant DNA technology and, hence, showed a high degree of purity (>99%) and quality attributes [1,3]. Highly purified human menopausal gonadotropin (HP-hMG) is noteworthy for the combination of FSH and LH activities. The bioactivity of LH in HP-hMG is assessed using the in vivo Steelman–Pohley rat bioassay which does not distinguish between LH activity contributed by LH or by hCG (pituitary or placental). Thus, the source of LH activity in HP-hMG has been debated, with distinct biological effects being associated with each source [2].

HP-hMG [Menopur^®^, Ferring Pharmaceuticals, Saint-Prex, Switzerland] is claimed to contain 1:1 FSH and LH activity, extracted from the urine of postmenopausal women, according to its summary of product characteristics [4]. After the development of a new purification process aimed at improving pharmaceutical quality of HP-hMG by reducing the presence of non-related gonadotropin proteins, as described in its patent [5], the manufacturer of HP-hMG stated that a purity of 95% and high batch-to-batch consistency was achieved [5,6,7]. However, Bassett et al. reported that HP-hMG preparations did not achieve a high degree of purity and non-gonadotropin proteins were detected at variable levels in different batches of HP-hMG [8]. In another analysis, Van der Weijer showed that up to 30% of HP-hMG samples consisted of impurities [9]. According to the manufacturer’s claims, the new purification procedure also reduced the pituitary hLH (pituitary human LH) contained in the final pharmaceutical preparation, while it concentrated the pituitary human chorionic gonadotropin (hCG) [5], compensating for the pituitary hLH lost. Regulatory bodies have stated that to compensate for the loss of pituitary LH during HP-hMG purification, it may be necessary to add urinary hCG extracted from the urine of pregnant women. This addition aims to achieve the desired 1:1 FSH:LH ratio, with a maximum of 20% of the total LH activity as measured by the in vivo Steelman–Pohley rat bioassay [10,11].

The LH receptor-mediated bioactivity of HP-hMG has been primarily attributed to naturally occurring hCG of pituitary origin in postmenopausal urine, adjusted in bioactivity with placental hCG for standardization [4]. However, it should be noted that there is considerable inconsistency in the manufacturer’s report of the source of LH activity and in the summary of product characteristics in different countries (UK, Australia, and Canada compared to Switzerland, Supplementary Table S1). The UK SmPC claims that the LH activity in HP-hMG was driven by naturally occurring pituitary hCG [4]. However, in May 2023, the manufacturers of HP-hMG reported unapproved changes in the production process to health authorities as per a communication by Ferring [12]. These changes included the balancing of the ratio of FSH:LH activity with the addition of highly purified urinary (placental) hCG.

Before the communication of the unapproved changes in the production process in May 2023, evidence from publications over two decades suggested that hCG in HP-hMG may be of an exogenous placental origin. Giudice et al. [2] found hCG content to be 10-fold higher than LH, while in 2003, van de Weijer et al. reported three times more immunoreactive hCG to be present than LH. The authors of the latter study concluded that 95% of the LH bioactivity in HP-hMG was due to the presence of exogenous hCG [9]. As the purity of the hMG preparation is increased, it has been shown that more LH molecules are preferentially lost [7] and more exogenous hCG needs to be added to return to the required FSH:LH ratio of 1:1 in the original product [9]. Amongst commercially available urinary hMG products [7], the less purified products (e.g., Pergonal^®^) have been shown to contain more endogenous LH when compared to more purified products like HP-hMG (Menopur^®^) where the majority of LH bioactivity has been provided by hCG supplementation [7].

Based on the evidence summarized above, the bioactivity of LH in HP-hMG may be contributed by LH, pituitary hCG, and/or placental hCG. The two glycoprotein hormones, hCG and LH, share a common alpha subunit, have highly homologous beta subunits, and bind to the same receptor [13]. However, 1 IU of placental hCG exerts an equivalent of 6 IU of LH activity, as assessed by the Steelman–Pohley in vivo rat bioassay [2]. During pregnancy, placental hCG is produced in high concentrations, stimulates the corpus luteum to produce progesterone, and acts to maintain pregnancy [14]. It also promotes the growth of cytotrophoblast cells and invasion by these cells. Pituitary hCG is produced in low concentrations during the menstrual cycle and is also present in the urine of postmenopausal women [14]. Still, its role has not been fully elucidated [15]. Pituitary hCG has a shorter half-life compared to placental hCG, is less potent, and is secreted in a pulsatile manner by the pituitary gland [13]. Thus, the clinical effect of LH activity from pituitary hCG compared to placental hCG and LH can be considerably different, and a clear identification of the source of hCG in HP-hMG is important. Different hCG isoforms can be distinguished by their glycosylation pattern—pituitary hCG is characterized by the expression of sulfated, rather than the more typical sialylated, oligosaccharides [13].

Considering the previous evidence before May 2023 [7,8], which suggests that the exogenous addition of placental hCG to HP-hMG provides a large proportion of its LH activity, the current study was carried out between 2020 and 2023 to assess gonadotropin (FSH, LH, and hCG) abundance in HP-HMG and its sources. Specifically, the aim was to identify the source of LH activity in HP-hMG (Menopur^®^, Ferring Pharmaceuticals, Saint-Prex, Switzerland) and whether it arises from either placental or pituitary hCG. We applied a multi-step approach of characterization aimed at the determination of specific features that can allow us to discriminate between the pituitary and placental isoforms. In addition, quality attributes of the product were tested, such as purity and degradation profile.

## 2. Results

### 2.1. Intact Molecule Analysis by LC

**Figure 1:**
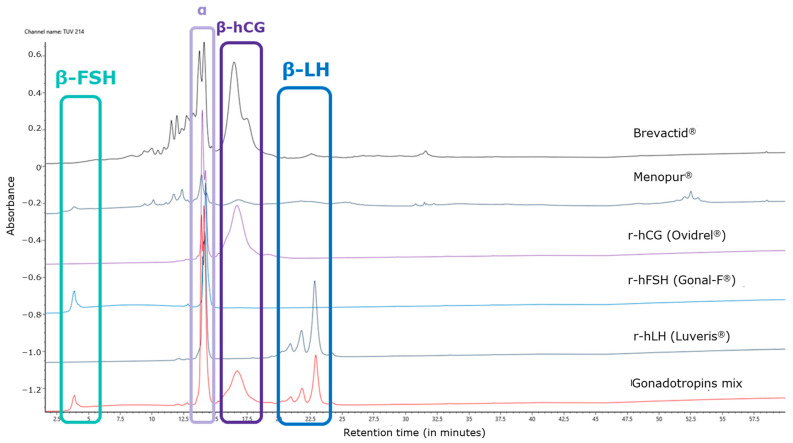
Overlay of UV profiles of tested HP-hMG, and products used as control samples for retention time and peak shapes. hCG, human chorionic gonadotropin; FSH, follicle-stimulating hormone; LC-MS, liquid chromatography–mass spectrometry; LH, luteinizing hormone; r-hFSH, recombinant human FSH; r-hLH, recombinant human LH; HP-hMG, highly purified human menopausal gonadotropin; UV, ultraviolet.

The chromatographic UV profile of the control allows us to attribute each subunit of each gonadotropin based on different elution times. The LH peak detected in the HP-hMG test sample was shown to be more prominent than expected. The alpha subunit was detected at a retention time of 14 min, while the beta subunits of FSH, hCG, and LH were detected at 4 min, 16 min, and 22 min, respectively. In particular, the peak shape of beta FSH is affected by the early elution in the chromatographic profile, while beta LH is characterized by a shape composed of three peaks, due to the C-terminal heterogeneity of the molecule (Figure 1). The oxidized alfa subunits of the gonadotropins eluted just before their alfa subunits during the LC.

**Figure 2:**
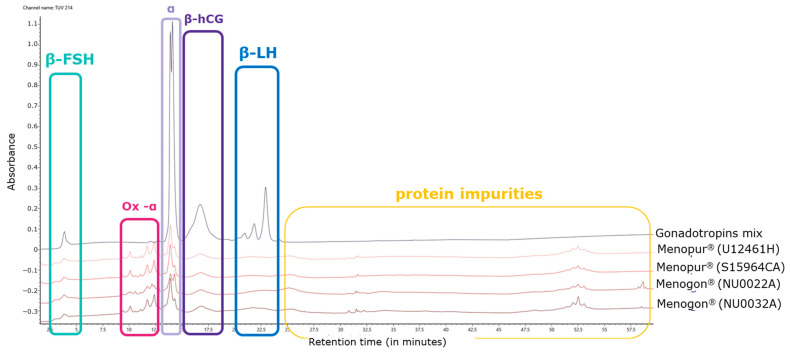
Overlay of UV profiles of HP-hMG test samples, together with the in-house gonadotropin mix. hCG, human chorionic gonadotropin; FSH, follicle-stimulating hormone; LH, luteinizing hormone; UV, ultraviolet. Menopur^®^ (Ferring Pharmaceuticals, Switzerland) (U12461H and SI5964CA)—Sample S15094CA was not used in this analysis; Menogon^®^ (Ferring Pharmaceuticals, Switzerland) (NU0022A and NU0032A).

Oxidized species related to the alpha subunit were detected in the range of 10–12 min and were confirmed by MS analysis. In particular, mono-, di-, and tri-oxidized species were identified in the HP-hMG test samples (highlighted in pink; Figure 2). Several proteins were detected in all HP-hMG test samples and were not observed in the control (gonadotropin mixture) sample, so they are probably associated with non-gonadotropin molecules (highlighted in yellow; Figure 2).

### 2.2. Sample Composition Assessed by Intact Molecule Analysis by LC-MS

The relative abundance of hCG in the HP-hMG test sample appears to be higher than that observed in the reference mix of gonadotropins. Additionally, the estimated amount of protein impurities in the preparation, determined by integration of UV profiles, was approximately 20–30% of the total protein content of the test sample. The relative abundance of the beta-hCG and beta-LH was compared within the test samples (Table 1). Further, the relative abundance of the sum of the mono-, di-, and tri-oxidized species was calculated as 20% of the total protein content in all the tested HP-hMG samples, and only 0.09% of the total protein content in the control sample with mixed recombinant gonadotropins (Figure 2 and Table 1).

### 2.3. Protein Impurity Analysis by LC-MS/MS

More than 200 protein impurities were identified in the samples (Table 2), including several proteins involved in embryonic development or female pregnancy, strongly suggesting that the hCG present in HP-hMG test samples is derived from an external placental source. Even in HP-hMG samples from a new liquid injectable formulation (NU0022A and NU0032A), nearly 100 protein impurities were found. In these HP-hMG test samples, when compared to the control samples of mixed recombinant gonadotropins, there was a lower relative abundance of LH compared to the relative abundance of hCG, which is in accordance with the preliminary estimation performed with the LC-MS/MS method (Table 3).

**Figure 3:**
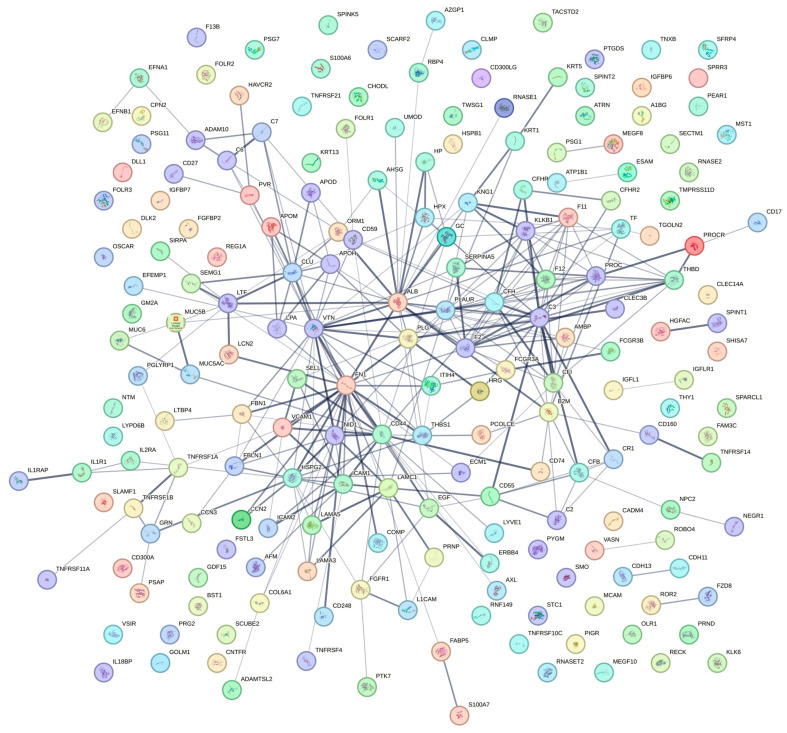
Protein impurity classification according to tissue expression and biological process. A complete list of the proteins presented above is presented in Supplementary Table S4.

The recalculated relative abundance of the main protein components was reported for all samples (Table 3). In order to understand the composition of gonadotropins in the tested HP-hMG samples, a parallel method was employed. In this method, the proportion of proteins was calculated using the MS profiles without taking into account all the protein impurities. Thus, the Brevactid^®^ library was not used in this method, and only the amounts of the three gonadotropins in the sample were calculated. In the tested HP-hMG samples, the beta subunit of LH was detected only in traces (0.9–1.2%), confirming the data obtained by intact molecule analysis, while high levels of beta hCG (18–47%) were observed (Table 3). An overview of the protein impurity classification according to tissue expression and biological process is presented in Figure 3. Characteristics of key identified protein impurities have been detailed in Supplementary Table S3 and a detailed list of the protein impurities can be found in Supplementary Table S4.

### 2.4. Reducing Peptide Mapping by LC-MS/MS

**Figure 4:**
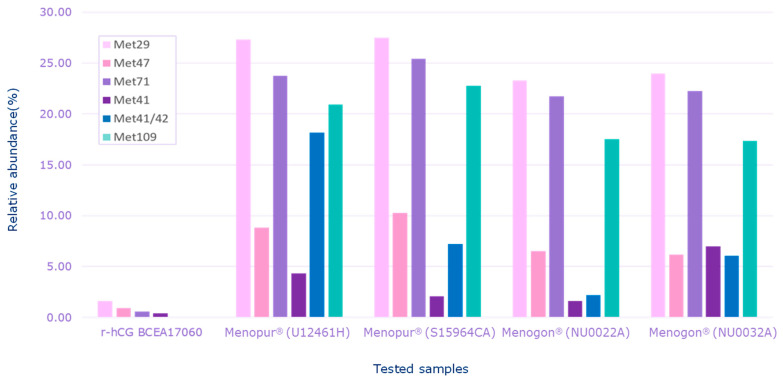
Relative abundance of oxidized species detected for each sample. Menopur^®^ (Ferring Pharmaceuticals, Saint Prex, Switzerland) (U12461H, S159464C); Menogon^®^ (Ferring Pharmaceuticals, Saint Prex, Switzerland) (NU0022A and NU0032A); Ovidrel^®^ (Merck KGaA, Darmstadt, Germany). r-hCG BCEA17060 was the recombinant human chorionic gonadotropin standard used as the reference sample. Met, methionine.

HP-hMG test samples were heavily oxidized in comparison to the recombinant hCG control sample (r-hCG BCEA17060), confirming the data shown by intact molecule analysis (Figure 4).

### 2.5. Glycopeptide Mapping by LC-MS/MS

The LH activity of the test HP-hMG sample appeared to be primarily due to hCG, as evidenced from the relative abundance of hCG and LH in the HP-hMG samples (Table 3). In the test HP-hMG sample, no traces of hCG with sulfated glycans were detected by glycopeptide mapping, indicating a potential absence of pituitary hCG and suggesting that placental hCG is likely the main source in analyzed samples.

#### 2.5.1. Alpha Subunit

**Figure 5:**
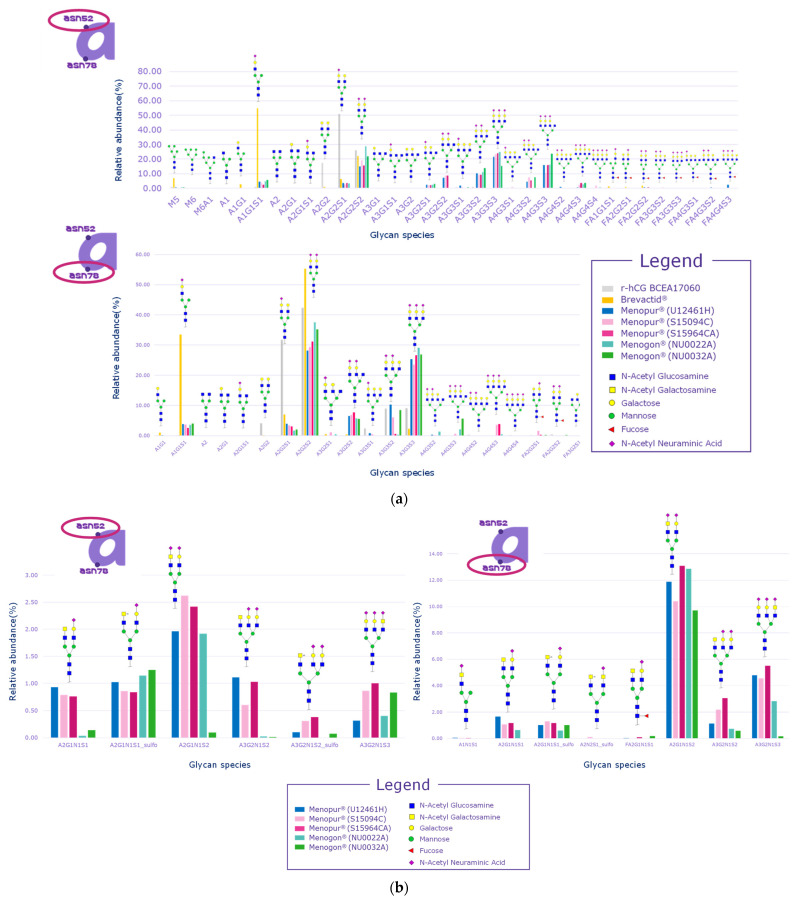
Glycan distribution on alpha subunits. (**a**) Glycan distribution for Asn52 and Asn 78; (**b**) sulfated glycan distribution for Asn52 and Asn 78. Menopur^®^ (Ferring Pharmaceuticals, Switzerland) (U12461H, S159094C, and S159464C); Menogon^®^ (Ferring Pharmaceuticals, Switzerland) (NU0022A and NU0032A); Ovidrel^®^ (Merck KGaA, Darmstadt, Germany). r-hCG BCEA17060 was the recombinant human chorionic gonadotropin standard used as the reference sample. The alphanumeric labels on the *x*-axis are the names of the species observed in the analysis and not acronyms.

Signals related to sulfated glycopeptides were detected on both Asn52 and Asn78 of the alpha subunit, which are justified by the pituitary source of FSH and LH in HP-hMG test samples. Indeed, signals relevant to sulfated glycans linked to N-glycosylation sites of the alpha subunit cannot be considered diagnostic, since all the gonadotropins share the same alpha subunit and it cannot be distinguished for LH, FSH, or hCG (Figure 5a,b). The distribution of glycans was observed to be comparable among the samples but different than the recombinant hCG control sample. Samples also had a different distribution of glycans compared to the urinary hCG (Brevactid^®^) control sample due to the presence of the alpha subunits of all three gonadotropins, while urinary hCG contains only the alpha subunit of hCG.

#### 2.5.2. Beta Subunits

**Figure 6:**
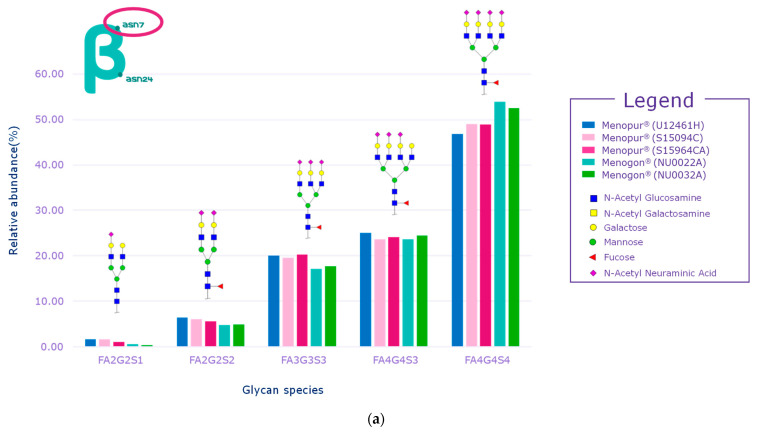

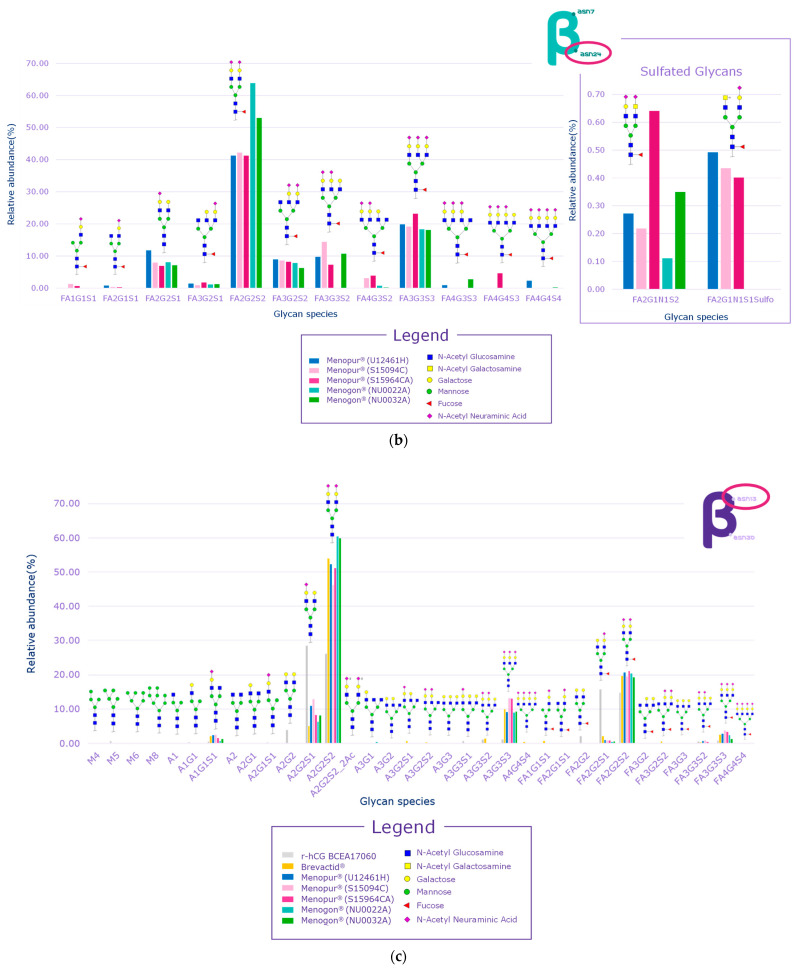

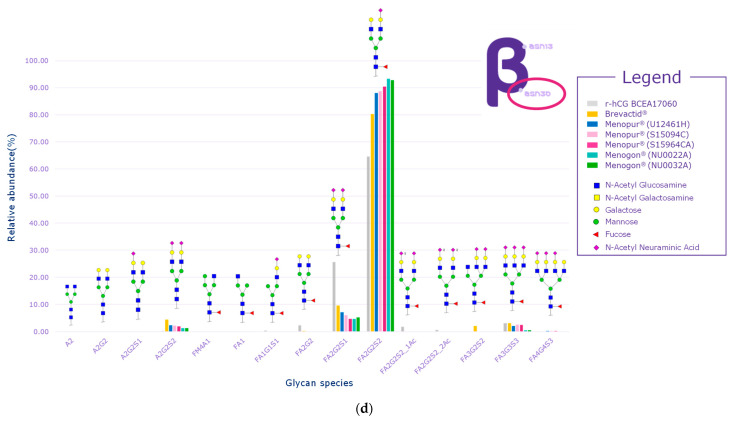
Glycan distribution of FSH/hCG beta subunit showing that the levels of glycans are consistent among the tested samples and that no sulfated glycans have been detected in the beta subunits of hCG. (**a**) Glycan distribution of FSH beta subunit—Asn7; (**b**) glycan distribution of FSH beta subunit—Asn24; (**c**) glycan distribution of hCG beta subunit—Asn13; (**d**) glycan distribution of hCG beta subunit—Asn30. Asn, asparagine; hCG, human chorionic gonadotropin; FSH, follicle-stimulating hormone. Three Menopur^®^ (Ferring Pharmaceuticals, Switzerland) (U12461H, S15094CA, and SI5964CA); Menogon^®^ (Ferring Pharmaceuticals, Switzerland) (NU0022A and NU0032A); Brevactid^®^ (Ferring Pharmaceuticals, Switzerland) (T14969E); Ovidrel^®^ (Merck KGaA, Darmstadt, Germany).

While several signals of sulfated glycans were detected in the beta subunit of the HP-hMG test samples, they pertained to glycopeptides derived from the beta subunit of pituitary FSH and trace levels of pituitary LH. The Asn7 of the beta subunit of FSH showed no traces of sulfated glycans, while signals related to GalNAc-GluNAc-containing species were detected at Asn24, confirming the pituitary source of FSH (Figure 6a,b). Only trace levels of sulfated glycans associated with LH were reported for some HP-hMG samples, probably due to the very low amount of LH in the preparation. The beta subunit of hCG showed no traces of sulfated glycans. Since no signals of sulfated glycopeptides were detected for any site of the beta subunit of hCG, it can be concluded that the hCG molecule in the HP-hMG sample is not from a pituitary source but from a placental source (Figure 6). It was also observed that HP-hMG test samples present the same glycan distribution as the urinary hCG sample, which is extracted from the urine of pregnant women, suggesting an addition from an external source of non-pituitary hCG (Figure 6). Further, no traces of signals related to the beta subunits of FSH and LH were found in the controls (r-hCG and u-hCG), validating our method’s performance.

## 3. Discussion

This study utilized a multi-step approach to assess gonadotropin (FSH, LH, and hCG) abundance in HP-hMG. Specifically, the aim was to identify the source of LH activity in HP-hMG and whether it arises from either placental or pituitary hCG. Quality attributes of the product, such as purity and degradation profile, were also tested. Evidence before May 2023 [7,8] suggested that the exogenous addition of placental hCG to HP-hMG provides a large proportion of its LH activity. The manufacturer of HP-hMG states that this formulation is composed of highly purified menotropin, which includes equal activities of human FSH and LH. The LH activity predominantly stems from hCG, a hormone naturally found in postmenopausal urine, as noted in the HP-hMG UK SmPC and supported by patent WO 15177751 [4,16]. To maintain a 1:1 ratio of FSH to LH activity, additional placental hCG, extracted from the urine of pregnant women, may be incorporated [10]. The Steelman–Pohley in vivo bioassay, recognized in both British and US pharmacopeias, assesses LH bioactivity in international units (IUs). This assay, as documented by Guidice et al. (2001) and Filicori et al. (1999), measures all sources of LH activity—pituitary LH, pituitary hCG, and placental hCG—without distinguishing between their origin [2,17].

Historical data suggest that a substantial part of the LH activity in HP-hMG originates from exogenous placental hCG. In their analysis, Giudice et al. (2001) observed a low LH content (1 EIA-IU/vial) compared to a higher hCG content (11 EIA-IU/vial). Their SDS-PAGE analysis revealed an undetectable beta subunit of LH, leading to the conclusion that the 1:1 in vivo bioactivity ratio of FSH to LH is predominantly due to hCG rather than LH [2]. Subsequent studies by van der Weijer (2003) and Wolfenson et al. (2005) supported these findings, noting a significantly higher presence of hCG compared to LH [7,9]. Bassett et al. (2009) further confirmed these observations across different batches, reinforcing the hypothesis that hCG was intentionally added rather than being co-purified from pituitary sources [8]. In 2010, Almeida et al. differentiated the alpha and beta subunits of FSH, LH, and hCG of various preparations. Based on the analysis, the authors concluded that hCG was added to the HP-hMG preparation [18]. Recent biochemical evaluations by Ricetti et al. (2017) on the beta subunits of hCG in HP-hMG identified several forms of hCG with distinct molecular masses, suggesting a placental origin due to the presence of a hyperglycosylated heterodimer [19].

In May 2023, the manufacturer notified health authorities that due to changes in HP-hMG production, the new primary source of hCG was the urine of pregnant women [12]. Our study, conducted prior to the manufacturer’s May 2023 announcement [12], aimed to verify the source of hCG in HP-hMG preparations. We found that the relative abundance of LH was significantly lower than that of hCG, with an absence of hCG sulfated glycans, ruling out a pituitary origin. These findings align with earlier studies [2,7,8], indicating that placental hCG was added to compensate for the lack of LH activity. Based on this previous published evidence and results presented in this study, it is reasonable to assume that placental hCG was added to compensate for the lack of LH-like activity due to the HP-hMG purification process introduced by the manufacturer in 2000 [5].

Furthermore, we assessed the product quality regarding non-gonadotropin protein contaminants and degradation products. The analysis revealed over 200 non-gonadotropin protein impurities, making up 20–30% of the total protein in HP-hMG products, contrasting with the high purity of recombinant preparations such as r-hFSH, r-hLH, and r-hCG [20,21,22,23,24]. This discrepancy highlights significant quality differences between recombinant and urine-derived products, previously documented in the literature [20,21]. The presence of numerous contaminants, some associated with embryonic development and pregnancy, supports the external placental source of hCG. This contradicts the manufacturer’s earlier claims of achieving 95% purity [5]. The analytical and quality differences between recombinant products and highly purified products of urinary origin have been previously highlighted in the literature [21]. Furthermore, Bassett et al. have shown the presence of 23 non-gonadotropin-related proteins at variable levels in different batches of the urine-derived gonadotropin preparations [8].

A recent publication reported that the administration of pituitary growth hormone contaminated with beta-amyloid protein led to the development of Alzheimer’s disease, underscoring the potential safety issues posed by protein impurities [25]. In the current analysis, two out of four HP-hMG test samples showed the presence of beta-amyloid. This finding, along with the consistent detection of significant contaminants over the past two decades, indicates minimal improvement in product quality [8,9,21]. Finally, HP-hMG exhibits a high degree of oxidized samples. Research has shown that oxidized gonadotropin samples exhibit lower biopotency in both in vivo and in vitro assays [26], potentially impacting clinical effectiveness.

The major barrier to improving the quality of urine-derived gonadotropins lies in the control of the raw material, which comprises pooled menopausal urine obtained from various individual contributors. Even small changes in the purification process will produce batches of urinary gonadotropins that will differ in the concentration and nature of each contaminant [8]. Such variability in the source and processing potentially contributes to the between-batch variation in impurities, particularly evident in urinary HP-hMG [8]. Furthermore, urine-derived gonadotropin preparations contain intact FSH dimers, inactive FSH forms, and contaminants, all of which contribute to the final mass, making it unfeasible to calculate its specific activity.

Lastly, the substitution of LH with hCG raises concerns about the clinical implications due to their different molecular and functional properties. Pituitary hCG, which is secreted in a pulsatile manner by the pituitary gland, is found in limited quantities in the blood of menopausal women [13]. The clinical implications of exogenous hCG supplementation in HP-hMG to achieve a 1:1 FSH:LH bioactivity ratio is unclear. While hCG can mimic the bioactivity of LH in the in vivo animal model, there are differences between LH and hCG at the molecular and functional level, as assessed in in vitro human models [27]. Recent studies have shown distinct signaling pathways activated by LH and hCG. LH primarily activates the ERK1/2-dependent and AKT-dependent pathways, while hCG primarily activates steroidogenic and pro-apoptotic pathways via PKA and CREB [19,27]. The unique molecular features of LH and hCG result in hormone-specific LHCGR binding and intracellular signaling cascades [28]. In ovarian cells, LH action is preferentially exerted through kinases, phosphorylated extracellular-regulated kinase 1/2 (pERK1/2), and phosphorylated AKT (protein kinase B), leading to essential proliferative/antiapoptotic signals and partial agonism on progesterone production in vitro [27,29]. In contrast, hCG demonstrates notable cAMP/protein kinase A (PKA)-mediated steroidogenic and proapoptotic potential, which is attenuated by estrogen action in vivo [29].

The results of the current study have been obtained using methods which reflect the most advanced techniques accepted by health authorities for full protein characterization. The corroboration of the results in these analyses by a multi-step approach is a key strength of this study. It must be noted that the methods have been developed and validated according to ICH criteria for recombinant products used as reference standards in this research and have not been validated specifically for HP-hMG. Nevertheless, this limitation can be partly offset by the fact that the methods have been validated for the same type of protein but from a recombinant origin. It should also be noted that a mixture of recombinant gonadotropins from one batch each was used as the reference sample for assessing purity when compared to HP-hMG. In our opinion, the use of one preparation of a mixture of recombinant gonadotropins from one batch each is enough for use as a reference standard for the assessment of impurities in the current analysis, as several previous papers have reported on the high level of purity of recombinant gonadotropins [1,30,31]. As such, the utilization of high-resolution physicochemical characterization methods significantly mitigates the likelihood of bias in the obtained results. Furthermore, the repetition of experiments using five independent samples of the same test molecule, HP-hMG, serves to further mitigate the risk of bias, thereby enhancing the overall reliability of the results. The results presented in this research stem from high-resolution analytical methods and accurately depict the quality of both the pharmaceutical preparation types analyzed, including both the test samples (HP-hMG) and the control samples used (recombinant gonadotropins).

Although the alpha subunits of LH and hCG show a high degree of similarity, the beta subunit is unique to each hormone. Apart from having an additional 24 amino acids, the beta chain of hCG possesses eight glycosylation sites, while LH has three [30]. The additional glycosylation sites give hCG a longer half-life. The terminal half-life via the subcutaneous route is 32–33 h for recombinant hCG vs. 21–24 h for r-hLH [32,33] and is characterized by a different LHCG-receptor activation [28,34]. It is the beta subunit of the hormone that confers its specificity and particular physiological activity [32]. However, considering the known differences in the biological and molecular properties of these molecules, the practice of substituting LH activity using hCG should be further studied for any clinical implication.

## 4. Materials and Methods

### 4.1. Samples Used

- Three batches of Menopur^®^ (hFSH and hLH; Ferring Pharmaceuticals, Saint-Prex, Switzerland) (batch number: U12461H, S15094CA, and SI5964CA) HP—freeze-dried.
- Two batches of Menogon^®^ (hFSH and hLH; Ferring Pharmaceuticals, Saint-Prex, Switzerland) (batch number: NU0022A and NU0032A) HP—PEN. Menogon is the same formulation as Menopur^®^ but is sold under a different brand name.
- One batch of Brevactid^®^ (hCG, Ferring Pharmaceuticals, Saint-Prex, Switzerland) (batch number: T14969E). Freeze-dried and contains urinary hCG extracted from the urine of pregnant women.
- One batch of Ovidrel^®^ (r-hCG, Merck KGaA, Darmstadt, Germany) (Batch BCEA17060).
- One batch of pituitary LH standard (Merck KGaA, Darmstadt, Germany) (Batch 869003).

In the current analyses, all the control samples used a single batch of the pharmaceutical product with the specific intention of identifying either the pattern of glycosylation suggestive of pituitary or placental hCG or for the identification of LH and hCG molecules. The control samples have been widely reported to contain the characteristics of interest (glycosylation pattern for pituitary origin or identification of LH and hCG) [21,30,31,35]. Five HP-hMG samples were used as the test samples to afford a more robust evaluation of HP-hMG. It should be noted that typically 3 batches is considered to be the minimum number necessary to validate the results obtained through an analytical method for a test sample. The test samples were constituted by pooling the HP-hMG contained in the vials or syringes based on the amount reported in IUs, in order to obtain the required amount of the drug for the analyzed samples.

For detailed information on the experimental considerations used for the different analyses, please refer to Supplementary Materials (methodology.pdf). An overview of the different test and control samples used in these analyses has been provided in Supplementary Table S2.

### 4.2. Glycopeptide Mapping by Hydrophilic Interaction Chromatography (HILIC)–Liquid Chromatography–Mass Spectrometry (LC-MS/MS)

Glycopeptide mapping enables differentiation between gonadotropin types by identifying glycans linked to specific peptides and the specific site of N-glycosylation unlike other techniques, like glycan mapping, which require glycan detachment from the molecule, losing the information about the original site of glycosylation. The complete glycan distribution profile of FSH, LH, and hCG were characterized in order to determine the contribution of all the components of the preparation. Samples were subjected to trypsin hydrolysis. The approach for the identification of hCG aimed to detect signals related to the presence of sulfated glycans, typical for pituitary species and not for the placental ones. The detection of sulfated glycan was used to confirm the presence of pituitary hCG. The presence of placental hCG was inferred by comparison of the glycan distribution of the test sample with that of the urinary hCG control (Brevactid^®^). The identity of glycopeptides was confirmed by fragmenting the peptide mixtures using high-collision dissociation (HCD). HCD fragmentation breaks the internal linkage of the glycans and gives two signals (the typical oxonium ions of 204.08 and 366.14, diagnostic for the presence of the N-acetylglucosamine and galactose), diagnostic of the glycopeptide moieties’ glycans. The commercially available HP-hCG preparation, Brevactid^®^, containing hCG extracted from the urine of pregnant women, was used as a negative control for comparing the distribution levels of glycan species. Recombinant hCG (Ovidrel^®^) was used as a control to compare the beta glycan distribution of the test samples with that of a recombinant hCG with a reported high purity. Pituitary LH standard (p-LH) was used as the control sample for the presence of sulfated glycans. Thyroid-stimulating hormone of bovine origin (b-TSH, Scripps laboratories) was used to optimize the presence of sulfated glycans.

A total of 25 vials of each batch of Menopur^®^, 2 syringes of each batch of Menogon^®^, and 2 vials of Brevactid^®^ 5000 IU were diluted in MilliQ water, pooled, and concentrated using Amicon Ultra 3K cartridges. For the sample preparation, 300 µg of each sample was treated, except for b-TSH and p-LH, for which 50 µg of sample was treated. Samples were diluted to the final concentration of 1 mg/mL with MilliQ water, and then, an equal volume of 8 M guanidine, 130 mM TRIS, and 1 mM EDTA (pH 7.6) was added. Samples were reduced by adding 30 µL (5 µL for b-TSH and p-LH) of DTT 500 mM (1 h, 37 °C) and then alkylated with 60 µL (10 µL for b-TSH and p-LH) of IAM 500 mM (1 h in the dark at ambient temperature). Each sample was washed five times with 200 µL of 2 M urea and 50 mM TRIS (pH 8.0) using Amicon Ultra 3K cartridges and then subjected to enzymatic hydrolysis. In particular, samples were subjected to hydrolysis with trypsin gold (Mass Spec grade, Promega) with an enzyme-to-substrate ratio of 1/10. Reactions were performed for 4 h at 37 °C and then stopped with 15 µL of 10% formic acid.

Samples were analyzed by UPLC–MS/MS on an Orbitrap Fusion Lumos (Thermo Fisher, Waltham, MA, USA) equipped with a Vanquish Horizon UPLC system by injecting 40 µL of the sample after dilution with 90% ACN in 0.02% TFA.

The data were processed using Expressionist software 13.0 (Genedata, Basel, Switzerland). The identification of N-glycans was performed on a single peptide per N-glycosylation site using a 50 ppm mass accuracy threshold followed by manual inspection of the mass spectra. Extracted ion chromatograms were used to calculate the relative abundance of each glycan per site.

### 4.3. Intact Molecule Analysis by Reversed-Phase (RP) Liquid Chromatography–Mass Spectrometry (LC-MS)

The HP-hMG test sample was analyzed using LC-MS carried out on a BioAccord System by Waters^®^, USA. This analysis also included a control mixture composed of r-hFSH/follitropin-alfa (GONAL-f^®^, Merck KGaA, Darmstadt, Germany), r-hLH/lutropin alfa (Luveris^®^, Merck KGaA, Darmstadt, Germany), and r-hCG (Ovidrel^®^, Merck KGaA, Darmstadt, Germany). The control, consisting of the recombinantly produced constituents, was used to assess the relative amount of the specific constituent and their retention time. The control was constituted to match the relative ratios of the three components based on their reported activity in the HP-hMG label. These recombinant gonadotropins are regarded as the reference standard in both pivotal and post-registration trials [36,37,38]. Their high quality and consistency have been rigorously tested, proven, and documented [20,21,30,31]. The results are presented as the relative abundance of each molecule, which “quantifies the amount of an ion produced in relation to the intensity of all the detected species” in each sample (ucla.edu). Thus, the abundance determined by UV light reflects the abundance of each analyzed component in the sample. The retention time of each species in the control is the time taken for a component in the sample to be detected.

The reference sample was obtained by mixing 50 µg of the three gonadotropin samples, r-hCG (Ovidrel^®^) 2018/01, r-hLH (Luveris^®^) 2019/01, and r-hFSH (Gonal-f^®^) 2016/01. In all, 20 µL of each sample at a concentration of 0.4 mg/mL was injected.

### 4.4. Analysis of Impurities by Reversed-Phase (RP) Liquid Chromatography–Mass Spectrometry LC-MS/MS

Impurities were profiled by LC-MS/MS to identify residual proteins coming from the purification of the samples. In particular, the analysis was focused on the determination of the source of impurities. The samples, analyzed in two independent replicates, were subjected to a procedure resulting in the generation of a peptide mixture which was then analyzed by LC-MS/MS. To enhance the sensitivity of the method, the positive control Brevactid^®^ (a known urinary hCG sample) was analyzed to build a library of peptides. The use of this approach leads to the generation of a library with detailed data on the specific peptides observed in an hCG sample of known placental origin. This library resulted in a more accurate peptide attribution by comparing the peptides in the test sample with those in the library, which in turn leads to a higher sensitivity in protein detection compared to an approach where a library is not generated. Phosphorylase B standard was used as an internal control. The Brevactid^®^ sample was first analyzed to build a protein library, employing a FASTA v36.3.8h database (a protein amino acid sequence alignment software) search limited to proteins of human origin. Proteins were then classified based on tissue expression and biological process. Proteins not part of the Brevactid^®^ library were also included in the search.

To each sample, a volume of solution of trypsin gold 1 mg/mL, equivalent to an enzyme-to-substrate ratio of 1:100, was added and incubated at 37 °C for 2 h. Then, 10 µL of a solution of 500 mM DTT in water was added and incubated for 10 min at 90 ˚C. At the end of the reaction, samples were centrifuged at 10,000 RPM for 5 min and the obtained supernatant was carefully transferred to a clean UPLC vial. Finally, 5 µL of a solution of 10% formic acid in MilliQ water was added to each sample to stop the hydrolysis reaction. The data were processed using Spectronaut (v 15.9) software (Biognosys, Schlieren, Switzerland).

### 4.5. Reducing Peptide Mapping by Reversed-Phase (RP) Liquid Chromatography–Mass Spectrometry (LC-MS/MS)

To investigate the nature of the degraded species previously detected in the profile of the intact molecule, reducing peptide mapping was performed, focusing on the characterization of the oxidized species. Reducing peptide mapping is an analytical method used to assess the primary structure of the molecule through the confirmation of the amino acid sequence and characterizing post-translational modifications (PTMs) by analyzing the molecular mass of detected species following chromatographic separation. Recombinant hCG was used as a control for this method to compare the oxidation levels of the generated peptides with those in the HP-hMG test sample. The results of this analysis are presented as graphical outputs of mass spectra for each sample with the peaks for relative abundance (in %) plotted against the retention time. The outputs for each of the samples are overlaid together for easy comparison of the results.

A total of 25 vials of each batch of Menopur^®^ and 2 syringes of each batch of Menogon^®^ were diluted in MilliQ water, pooled, and concentrated using Amicon Ultra 3K cartridges. For the sample preparation, 300 µg of each sample was diluted to the final concentration of 1 mg/mL with MilliQ water, and then, an equal volume of 8 M guanidine, 130 mM TRIS, and 1 mM EDTA (pH 7.6) was added. Samples were reduced by adding 30 µL of DTT 500 mM (1 h, 37 °C) and then alkylated with 60 µL of IAM 500 mM (1 h in the dark). Each sample was washed five times with 200 µL of 2 M urea and 100 mM TRIS (pH 8.0) using Amicon Ultra 3K cartridges and then subjected to enzymatic hydrolysis. In particular, samples were subjected to hydrolysis with trypsin gold with an enzyme-to-substrate ratio of 1:10. Reactions were performed for 4 h at 37 °C and then stopped with 15 µL of 10% formic acid.

## 5. Conclusions

Advanced molecular analysis of HP-hMG indicates a primarily placental origin of hCG, as evidenced by the absence of hCG sulfated glycans and the predominance of placental non-sulfated hCG in LH activity. The analysis of HP-hMG samples also revealed that total gonadotropin content constitutes only 70–80% of the total protein, with 20–30% comprising protein impurities, and there is a significant presence of oxidized forms. Without considering protein impurities, the relative abundance of LH was very low (confirming intact molecule analysis) and of hCG was high in all tested HP-HMG samples. These findings are critical for understanding the quality, safety, and clinical profile of HP-hMG.

## Figures and Tables

**Table 1:** Relative abundance of detected species calculated by integration of UV profiles (% of detected gonadotropins relative to the total protein content in the sample).

Species	Control Sample with Mixed Recombinant Gonadotropins	HP-hMG Test Samples			
		U12461H	SI5964CA	NU0022A	NU0032A
Beta hCG	26.04	11.44	12.26	10.27	11.37
Beta LH	20.63	10.57	6.69	4.96	5.95
Impurities	N.D.	27.71	22.76	32.23	31.53
Oxidized species (alpha subunit)	0.09	20.02	18.98	20.89	17.32

hCG, human chorionic gonadotropin; FSH, follicle-stimulating hormone; LC-MS, liquid chromatography–mass spectrometry; LH, luteinizing hormone; UV, ultraviolet. Menopur^®^ (Ferring Pharmaceuticals, Switzerland) (U12461H and SI5964CA)—Sample S15094CA was not used in this analysis; Menogon^®^ (Ferring Pharmaceuticals, Switzerland) (NU0022A and NU0032A).

**Table 2:** Protein impurity determination.

Samples	Recombinant hCG (Ovidrel^®^) Batch BCEA17060		HP-hMG Test Samples							
			U12461H		SI5964CA		NU0022A		NU0032A	
Replicates	Rep. 1	Rep. 2	Rep. 1	Rep. 2	Rep. 1	Rep. 2	Rep. 1	Rep. 2	Rep. 1	Rep. 2
Number of identified proteins	2	5	198	199	200	202	102	91	99	99

Sample S15094CA was not used in this analysis. Menopur^®^ (Ferring Pharmaceuticals, Switzerland) (U12461H, S15094CA); Menogon^®^ (Ferring Pharmaceuticals, Switzerland) (NU0022A and NU0032A); Ovidrel^®^ (Merck KGaA, Darmstadt, Germany).

**Table 3:** Relative abundance of gonadotropins (% of detected gonadotropins relative to the total protein content in the sample).

Species	Recombinant hCG (Ovidrel^®^) Batch BCEA17060	HP-hMG Test Sample			
		U12461H	SI5964CA	NU0022A	NU0032A
Alpha	26.28	43.00	50.27	32.65	50.33
Beta hCG	73.73	22.33	16.93	47.38	18.03
Beta FSH	N.A.	33.59	31.57	19.05	30.46
Beta LH	N.A.	1.09	1.25	0.92	1.19

N.A. not applicable (FSH and LH are not components of the negative control BCEA17060). Sample S15094CA was not used in this analysis. Menopur^®^ (Ferring Pharmaceuticals, Switzerland) (U12461H, S15094CA); Menogon^®^ (Ferring Pharmaceuticals, Switzerland) (NU0022A and NU0032A); Ovidrel^®^ (Merck KGaA, Darmstadt, Germany).

## Data Availability

Any requests for data by qualified scientific and medical researchers for legitimate research purposes will be subject to Merck KGaA’s Data Sharing Policy. All requests should be submitted in writing to Merck KGaA’s data sharing portal (https://www.merckgroup.com/en/research/our-approach-to-research-and-development/healthcare/clinical-trials/commitment-responsible-data-sharing.html; [accessed 25 June 2023]). When Merck KGaA has a co-research, co-development, or co-marketing or co-promotion agreement, or when the product has been out-licensed, the responsibility for disclosure might be dependent on the agreement between parties. Under these circumstances, Merck KGaA will endeavor to gain agreement to share data in response to requests.

## Supplementary Materials

The following supporting information can be downloaded at https://www.mdpi.com/article/10.3390/ijms25179405/s1.
