# Supplementary material for: Analytical Investigation of the Profile of Human Chorionic Gonadotropin in Highly Purified Human Menopausal Gonadotrophin Preparations
Source: Int J Mol Sci. 2024 Aug 29;25(17):9405. doi: 10.3390/ijms25179405 (PMC11395176; doi:10.3390/ijms25179405)
Supplement: Supplementary file 1 [file ijms-25-09405-s001.zip › Supplementary information_Methodology.pdf]

Experimental parameters for the analyses performed in this study

1.1. Glycopeptide analysis by LC-MS/MS

Table 1. UPLC Experimental conditions - Glycopeptide mapping by HILIC-LC-MS/MS

**Instrument**	Orbitrap Fusion Lumos (Thermo) equipped with an Vanquish Horizon UPLC
**Column**	Acquity UPLC BEH Amide column 130 Å (glycan) 1.7μm 2.1x150mm
**Flow rate**	0.2 mL/min
**Injection Volume**	5 μL
**Column Temperature**	50 °C ± 1 °C
**Autosampler Temperature**	5 °C ± 3 °C
**Eluents**	A: 0.1% TFA in H2O MilliQ<br>B: 0.1% 0.1% TFA in ACN
**UV detector**	214 nm
**Run Time**	94 min

Table 2. UPLC gradient – Glycopeptide mapping by HILIC-LC-MS/MS

Time (min)	B (%)	Curve
0.0	90	Initial
3.0	90	6
3.1	70	6
8.0	70	6
71.0	45	6
71.1	20	6
76.0	20	6
76.1	90	6
94.0	90	6

Table 1. MS Source Setting – Glycopeptide mapping by HILIC-LC-MS/MS

**Ion Source Type**	Heated ESI
**Positive Ion Spray Voltage (V)**	3500
**Sheath Gas (Arb)**	35
**Aux Gas (Arb)**	10
**Sweep Gas (Arb)**	0
**Ion Transfer Tube Temp (°C)**	325
**Vaporizer Temp (°C)**	275

Table 4. MS analyzer parameters – Glycopeptide mapping by HILIC-LC-MS/MS

**Intact Protein Mode**	False

**Polarity**	Positive
**Detector**	Orbitrap
**Orbitrap Resolution**	30000
**Use Quadrupole Isolation**	True
**Maximum Injection Time (s)**	50
**Scan range (m/z)**	350-3000
**RF lens (%)**	60
**AGC Target**	4e5
**Microscans**	1
**Include charge state**	2-7
**Dynamic Exclusion – exclude after n times**	1
**Dynamic Exclusion – exclusion duration (s)**	7
**Expected peak width (FWHM, s)**	10
**Desired Apex Window (%)**	30
**DDA mode**	Top S (3 sec)
**Activation Type**	HCD
**HCD Collision Energy**	Stepped – 15,20,28
**Detector Type**	Orbitrap
**Resolution**	15000
**Scan Range Mode**	Auto
**AGC Target**	1e5
**Maximum Injection Time (s)**	150
**Data Type**	Profile
**Source Fragmentation**	False
**Energy (V)**	False

1.2. Impurities analysis by RP-LC-MS/MS

Table 5. UPLC Experimental conditions – Impurities analysis by RP-LC-MS/MS

**Instrument**	Q-Exactive HFX (Thermo) equipped with an Vanquish Horizon UPLC
**Column**	Acquity UPLC Peptide CSH C18, 130 Å ,1.7 µm, 2.1 x 150 mm
**Flow rate**	0.2 mL/min
**Injection Volume**	70 µL
**Column Temperature**	60.0 ˚C ± 2.0 ˚C
**Autosampler Temperature**	5.0 ˚C ± 3.0 ˚C
**Eluents**	A: 0.1% formic acid in H_2O<br>B: 0.1% formic acid in acetonitrile
**UV detector**	214 nm

Table 6. UPLC gradient – Impurities analysis by RP-LC-MS/MS

Time (min)	Flow (mL/min)	%A	%B
0.0	0.20	99.0	1.0

Time (min)	Flow (mL/min)	%A	%B
2.0	0.20	99.0	1.0
92.0	0.20	45.0	55.0
94.0	0.20	45.0	55.0
95.0	0.20	10.0	90.0
97.0	0.20	10.0	90.0
9800	0.20	99.0	1.0
120.0	0.20	99.0	1.0

Table 7. MS conditions for library building - Impurities analysis by RP-LC-MS/MS

**Positive Ion (V)**	3500
**Negative Ion (V)**	3100
**Sheath Gas (Arb)**	45
**Aux Gas (Arb)**	10
**Sweep Gas (Arb)**	2
**Capillary temp (°C)**	250
**Funnel RF Level**	40
**Aux gas heater temp**	400
**Acquisition mode**	DIA
**Full scan**	
**MS Scan range (m/z)**	390-1050
**Orbitrap Resolution**	12000
**AGC Target**	3.0e6
**Maximum Injection Time (s)**	60
**MS/MS**	
**Activation Type**	HCD
**Detector Type (MS/MS)**	Orbitrap
**Orbitrap Resolution (MS/MS)**	30000
**AGC target**	1.0e6
**Isolation window (m/z)**	4.0
**HCD Collision Energy (%)**	27
**Maximum Injection Time (s)**	60
**MS precursors range (m/z)**	400-900 with 100 m/z per run (i.e. first run 400-500, second run 500-600 etc. with an overlap of 0.5 m/z)

Table 8. MS condition for DS samples – Impurities analysis by RP-LC-MS/MS

**Positive Ion (V)**	3500
**Negative Ion (V)**	3100
**Sheat Gas (Arb)**	45

**Aux Gas (Arb)**	10
**Sweep Gas (Arb)**	2
**Capillary temp (°C)**	250
**Funnel RF Level**	60
**Aux gas heater temp**	400
**Acquisition mode**	DIA
**Full scan**	
**MS Scan range (m/z)**	400-900
**Orbitrap Resolution**	60000
**AGC Target**	3.0e6
**Maximum Injection Time (s)**	65
**Inclusion list**	359.0, 376.5, 394.0, 411.5, 429.0, 446.5, 464.0, 477.5, 487.0, 496.5, 506.0, 515.5, 525.0, 534.5, 544.0, 553.5, 563,0 572.5, 582.0, 591.5, 601.0, 610.5, 620.0, 629.5, 639.0, 648.5, 658.0, 667.5, 677.0, 686.5, 696.0, 705.5, 715.0, 724.5, 734.0, 743.5, 753.0, 762.5, 772.0, 781.5, 791.0, 800.5, 814.0, 831.5, 849.0, 866.5, 884.0
**MS/MS**	
**Activation Type**	HCD
**Detector Type (MS/MS)**	Orbitrap
**Orbitrap Resolution (MS/MS)**	30000
**AGC target**	1.0e5
**Isolation window (m/z)**	18,10
**HCD Collision Energy (%)**	27
**Maximum Injection Time (s)**	60

The data were processed using Spectronaut software (Biognosys).

1.3. Intact Molecule analysis by RP-LC-MS

Table 9. UPLC Experimental conditions – Intact Molecule analysis by RP-LC-MS

**Instrument**	BioAccord (Waters) coupled with Acquity Premier UPLC
**Column**	Acquity UPLC Protein BEH C4 Column, 300Å, 1.7 µm, 2.1 mm X 50 mm
**Flow rate**	0.4 mL/min
**Injection Volume**	20 µL
**Column Temperature**	35 °C ± 1 °C
**Autosampler Temperature**	5 °C ± 3 °C
**Eluents**	A: 0.1% TFA in H2O MilliQ<br>B: 0.1% TFA in ACN
**UV detector**	214 nm

**Run Time**	87 min

Table 2 UPLC gradient – Intact Molecule analysis by RP-LC-MS

Time (min)	B (%)	Curve
0.0	15	Initial
40.0	40	6
45.0	40	6
65.0	70	6
66.0	90	6
76.0	90	6
77.0	15	6
87.0	15	6

Table 3. MS Source Setting – Intact Molecule analysis by RP-LC-MS

**Capillary Voltage (kV)**	1.50
**Desolvation Temp (°C)**	450
**Cone Voltage (V)**	35

Table 4 MS analyzer parameters - Intact Molecule analysis by RP-LC-MS

**Polarity**	Positive
**Detector**	TOF
**Scan range (m/z)**	400-7000

1.4. Reducing peptide mapping by RP-LC-MS/MS

Table 13. UPLC experimental conditions – Reducing peptide mapping by RP-LC-MS/MS

**Column**	Acquity UPLC Peptide CSH C18, 130 Å ,1.7 µm, 2.1 x 150 mm
**Flow rate**	0.4 mL/min
**Injection Volume**	5 µL
**Column Temperature**	50 °C ± 1 °C
**Autosampler Temperature**	5 °C ± 3 °C
**Eluents**	A: 0.1% formic acid in MilliQ water<br>B: 0.1% formic acid in ACN
**UV detector**	214 nm
**Run Time**	min

Table 14. UPLC gradient – Reducing peptide mapping by RP-LC-MS/MS

Time (min)	B (%)	Curve
0.0	1	Initial

2.0	1	6
8.0	7	6
13.0	12	6
40.0	35	6
41.0	90	6
46.0	90	6
46.1	1	6
50.0	1	6

Table 15. MS experimental conditions – Reducing peptide mapping by RP-LC-MS/MS

**Positive Ion (V)**	3500
**Sheat Gas (Arb)**	50
**Aux Gas (Arb)**	12.5
**Sweep Gas (Arb)**	2
**Capillary temp (°C)**	260
**Funnel RF Level**	60
**Aux gas heater temp**	425
**Acquisition mode**	DDA
**Full scan**	
**MS Scan range (m/z)**	200-2000
**Orbitrap Resolution**	60000
**AGC Target**	1.0e6
**Maximum Injection Time (s)**	150
**MS/MS**	
**Activation Type**	HCD
**Detector Type (MS/MS)**	Orbitrap
**Orbitrap Resolution (MS/MS)**	30000
**AGC target**	5.0e4
**HCD Collision Energy (%)**	28
**Maximum Injection Time (s)**	150
