# Supplementary material for: Analytical Investigation of the Profile of Human Chorionic Gonadotropin in Highly Purified Human Menopausal Gonadotrophin Preparations
Source: Int J Mol Sci. 2024 Aug 29;25(17):9405. doi: 10.3390/ijms25179405 (PMC11395176; doi:10.3390/ijms25179405)
Supplement: Supplementary file 1 [file ijms-25-09405-s001.zip › Supplementary table S1.pdf]

Supplementary table S1. Summary of the SmPC claims about the source of hCG in HP-hMG in different countries

Country	SmPC text
**Menopur 75 IU UK SmPC (updated November 2019)**	*Section 2*: Each vial of powder contains highly purified menotrophin (human menopausal gonadotrophin, hMG) corresponding to 75 IU human follicle stimulating hormone (FSH) and 75 IU human luteinising hormone (LH) activity. Human Chorionic Gonadotrophin (hCG), a naturally occurring hormone in postmenopausal urine, is present in MENOPUR® and contributes to the overall luteinizing hormone activity. Menotrophin is produced from human urine. *Section 5.1*: Menotrophin (Human Menopausal Gonadotrophin, hMG) is a gonadotrophin extracted from the urine of postmenopausal women. It has both luteinising hormone and follicle stimulating hormone activity in a 1:1 ratio. Human Chorionic Gonadotrophin (hCG), a naturally occurring hormone in postmenopausal urine, is present in MENOPUR and is the main contributor of the LH activity.
**Menopur pen Swiss SmPC (April 2021)**	Menotropin, from human urine, country of origin of urine: Argentina. *Menopur 75 IU vial*: 1 vial of powder contains: human follicle stimulating hormone (FSH) 75 IU, human luteinizing hormone (LH) 75 IU. *Menopur Multidose Pen 600 IU*: 1 prefilled pen contains: 600 IU menotropin. Per ml, the reconstituted solution contains 600 IU of FSH and LH. The concentration of the injection solution in the pen is 625 IU/ml. *Mode of action*: Menopur consists of highly purified human menopausal gonadotropin (hMG), a hormone mixture composed of follicle stimulating hormone (FSH) and luteinizing hormone (LH) in a ratio of approximately 1:1. Menopur consists of highly purified, human menopausal gonadotropin (hMG), a hormone mixture composed of follicle-stimulating hormone (FSH) and luteinising hormone (LH) at a ratio of approximately 1:1. The significantly longer biological half-life of FSH compared to LH means that Menopur primarily has an FSH effect.
**Canada Product Monograph**	Menopur® is produced from urine of postmenopausal women. Human Chorionic Gonadotropin (hCG), a naturally occurring hormone in postmenopausal women, is present in Menopur® and contributes to the overall luteinizing hormone (LH) activity.
**Australia Product Information**	Human Chorionic Gonadotrophin (hCG), a naturally occurring hormone in postmenopausal urine, is present in MENOPUR and is the main contributor of the LH activity. MENOPUR, which contains both FSH and LH activity, induces ovarian follicular growth and development as well as gonadal steroid production in women who do not have primary ovarian failure. FSH is the primary driver of follicular recruitment and growth in early folliculogenesis, while LH is important for ovarian steroidogenesis and is involved in the physiological events leading to the development of a competent pre-ovulatory follicle. Follicular growth can be stimulated by FSH in the total absence of LH, but the resulting follicles develop abnormally and are associated with low oestradiol levels and inability to luteinise to a normal ovulatory stimulus.
