# Supplementary material for: Analytical Investigation of the Profile of Human Chorionic Gonadotropin in Highly Purified Human Menopausal Gonadotrophin Preparations
Source: Int J Mol Sci. 2024 Aug 29;25(17):9405. doi: 10.3390/ijms25179405 (PMC11395176; doi:10.3390/ijms25179405)
Supplement: Supplementary file 1 [file ijms-25-09405-s001.zip › Supplementary table S2.pdf]

Supplementary table S2. Summary of the various analyses and samples used

Sample	Glycopeptide mapping by LC-MS/MS	Intact molecule analysis by LC-MS	Analysis of protein impurities by LC-MS/MS	Reducing peptide mapping by LC-MS/MS
Menopur®/ Menogon®	Test	Test	Test	Test
Ovidrel® (r-hCG-BCEA17060)	**Control** To compare the test sample with the beta glycan distribution of a recombinant hCG of high purity	Not used in the analysis	**Control** To compare the test sample with a recombinant hCG of high purity	**Control** To compare the levels of oxidized peptides
Reconstituted r-hCG (Ovidrel®) - DS, r-LH (Luveris®) - DS, and r-FSH (Gonal F®) DS	Not used in the analysis	**Control** To compare retention times of the test sample with a mixture of individual recombinant products	Not used in the analysis	Not used in the analysis
Brevactid®	**Control** To compare the test sample with the beta glycan distribution of a urinary (placental) hCG	**Control** To compare the retention times of the test sample with a known urinary (placental) hCG sample	**Control** To compare the test sample with a known urinary (placental) hCG sample	Not used in the analysis
p-LH	**Control** To compare the test sample for the presence of sulfated glycan	Not used in the analysis	Not used in the analysis	Not used in the analysis

FSH, follicle stimulating hormone; hCG, human chorionic gonadotropin; p-LH, pituitary luteinizing hormone; LC-MS/MS, liquid chromatography-tandem mass spectrometry; LC-MS, liquid chromatography mass spectrometry.
