# Supplementary material for: Analytical Investigation of the Profile of Human Chorionic Gonadotropin in Highly Purified Human Menopausal Gonadotrophin Preparations
Source: Int J Mol Sci. 2024 Aug 29;25(17):9405. doi: 10.3390/ijms25179405 (PMC11395176; doi:10.3390/ijms25179405)
Supplement: Supplementary file 1 [file ijms-25-09405-s001.zip › Supplementary table S3.pdf]

Supplementary table S3. Characteristics of key protein impurities identified in the test samples

Gene	Protein	Function and Expression (STRING database)
PSG1/PSG11	Pregnancy-specific beta-1-glycoprotein	PSBG are produced in high quantity during pregnancy.
FOLR1/FOLR2	Folate receptor	Required for normal embryonic development and normal cell proliferation, those receptors are responsible for binding to folic acid and its derivatives, which becomes crucial during fetal development.
TWSG1	Twisted gastrulation protein homolog 1	Expressed in brain throughout development. It is also a reported modulator of BMP-regulated cartilage development and chondrocyte differentiation.
FZD2	Frizzled-2	Involved in transduction and intercellular transmission of polarity information during tissue morphogenesis and/or in differentiated tissues.
TNXB	Tenascin-X	Highly expressed in fetal adrenal, in fetal testis, fetal smooth, striated and cardiac muscle
LAMC1/LAMA5	Laminin subunit	Binding to cells via a high affinity receptor, laminin is thought to mediate the attachment, migration and organization of cells into tissues during embryonic development by interacting with other extracellular matrix components.
GDF15	Growth/differentiation factor 15	Highly expressed in placenta, with lower levels in prostate and colon and some expression in kidney. Acts as a pleiotropic cytokine and is involved in the stress response program of cells after cellular injury
PRG2	Proteoglycan 2	High levels of the proform of this protein are present in placenta and pregnancy serum. May be involved in antiparasitic defense mechanisms as a cytotoxin and helminthotoxin, and in immune hypersensitivity reactions
FBLN1	Fibulin-1	Widely expressed during embryonic development. May play a role in cell adhesion and migration along protein fibers within the extracellular matrix
