# Supplementary material for: Analytical Investigation of the Profile of Human Chorionic Gonadotropin in Highly Purified Human Menopausal Gonadotrophin Preparations
Source: Int J Mol Sci. 2024 Aug 29;25(17):9405. doi: 10.3390/ijms25179405 (PMC11395176; doi:10.3390/ijms25179405)
Supplement: Supplementary file 1 [file ijms-25-09405-s001.zip › Supplementary table S4.pdf]

Supplementary Table S4. Non-gonadotropin protein impurities found in HP-hMG test samples

PG.ProteinGroups	PG.Genes	PG.ProteinDescriptions
**P04217**	A1BG	Alpha-1B-glycoprotein
**O14672**	ADAM10	Disintegrin and metalloproteinase domain-containing protein 10
**Q86TH1**	ADAMTSL2	ADAMTS-like protein 2
**P43652**	AFM	Afamin
**P02765**	AHSG	Alpha-2-HS-glycoprotein
**P02768**	ALB	Albumin
**P02760**	AMBP	Protein AMBP
**P05090**	APOD	Apolipoprotein D
**P02749**	APOH	Beta-2-glycoprotein 1
**O95445**	APOM	Apolipoprotein M
**P05026**	ATP1B1	Sodium/potassium-transporting ATPase subunit beta-1
**O75882**	ATRN	Attractin
**P30530**	AXL	Tyrosine-protein kinase receptor UFO
**P25311**	AZGP1	Zinc-alpha-2-glycoprotein
**P61769**	B2M	Beta-2-microglobulin
**Q10588**	BST1	ADP-ribosyl cyclase/cyclic ADP-ribose hydrolase 2
**P06681**	C2	Complement C2
**P13671**	C6	Complement component C6
**P10643**	C7	Complement component C7
**Q8NFZ8**	CADM4	Cell adhesion molecule 4
**P29279**	CCN2	CCN family member 2
**P48745**	CCN3	CCN family member 3
**O95971**	CD160	CD160 antigen
**Q8N6Q3**	CD177	CD177 antigen
**Q9HCU0**	CD248	Endosialin
**P26842**	CD27	CD27 antigen
**Q9UGN4**	CD300A	CMRF35-like molecule 8
**Q6UXG3**	CD300LG	CMRF35-like molecule 9
**P16070**	CD44	CD44 antigen
**P08174**	CD55	Complement decay-accelerating factor
**P13987**	CD59	CD59 glycoprotein
**P04233**	CD74	HLA class II histocompatibility antigen gamma chain
**P55287**	CDH11	Cadherin-11
**P55290**	CDH13	Cadherin-13
**P00751**	CFB	Complement factor B
**P08603**	CFH	Complement factor H

**Q03591**	CFHR1	Complement factor H-related protein 1
**P36980**	CFHR2	Complement factor H-related protein 2
**P05156**	CFI	Complement factor I
**Q9H9P2**	CHODL	Chondrolectin
**Q86T13**	CLEC14A	C-type lectin domain family 14 member A
**P05452**	CLEC3B	Tetranectin
**Q9H6B4**	CLMP	CXADR-like membrane protein
**P10909**	CLU	Clusterin
**P26992**	CNTFR	Ciliary neurotrophic factor receptor subunit alpha
**P12109**	COL6A1	Collagen alpha-1(VI) chain
**P49747**	COMP	Cartilage oligomeric matrix protein
**P22792**	CPN2	Carboxypeptidase N subunit 2
**P17927**	CR1	Complement receptor type 1
**Q6UY11**	DLK2	Protein delta homolog 2
**O00548**	DLL1	Delta-like protein 1
**Q16610**	ECM1	Extracellular matrix protein 1
**Q12805**	EFEMP1	EGF-containing fibulin-like extracellular matrix protein 1
**P20827**	EFNA1	Ephrin-A1
**P98172**	EFNB1	Ephrin-B1
**P01133**	EGF	Pro-epidermal growth factor
**Q15303**	ERBB4	Receptor tyrosine-protein kinase erbB-4
**Q96AP7**	ESAM	Endothelial cell-selective adhesion molecule
**P03951**	F11	Coagulation factor XI
**P00748**	F12	Coagulation factor XII
**P05160**	F13B	Coagulation factor XIII B chain
**P00734**	F2	Prothrombin
**Q01469**	FABP5	Fatty acid-binding protein 5
**Q92520**	FAM3C	Protein FAM3C
**P23142**	FBLN1	Fibulin-1
**P35555**	FBN1	Fibrillin-1
**P08637**	FCGR3A	Low affinity immunoglobulin gamma Fc region receptor III-A
**O75015**	FCGR3B	Low affinity immunoglobulin gamma Fc region receptor III-B
**Q9BYJ0**	FGFBP2	Fibroblast growth factor-binding protein 2
**P11362**	FGFR1	Fibroblast growth factor receptor 1
**P02751**	FN1	Fibronectin
**P15328**	FOLR1	Folate receptor alpha
**P14207**	FOLR2	Folate receptor beta
**P41439**	FOLR3	Folate receptor gamma
**O95633**	FSTL3	Follistatin-related protein 3
**O75084;Q14332**	FZD7;FZD2	Frizzled-7;Frizzled-2
**Q9H461**	FZD8	Frizzled-8

**P02774**	GC	Vitamin D-binding protein
**Q99988**	GDF15	Growth/differentiation factor 15
**P17900**	GM2A	Ganglioside GM2 activator
**Q8NBJ4**	GOLM1	Golgi membrane protein 1
**P28799**	GRN	Progranulin
**Q8TDQ0**	HAVCR2	Hepatitis A virus cellular receptor 2
**Q04756**	HGFAC	Hepatocyte growth factor activator
**P00738**	HP	Haptoglobin
**P02790**	HPX	Hemopexin
**P04196**	HRG	Histidine-rich glycoprotein
**P04792**	HSPB1	Heat shock protein beta-1
**P98160**	HSPG2	Basement membrane-specific heparan sulfate proteoglycan core protein
**P05362**	ICAM1	Intercellular adhesion molecule 1
**P13598**	ICAM2	Intercellular adhesion molecule 2
**P24592**	IGFBP6	Insulin-like growth factor-binding protein 6
**Q16270**	IGFBP7	Insulin-like growth factor-binding protein 7
**Q6UW32**	IGFL1	Insulin growth factor-like family member 1
**Q9H665**	IGFLR1	IGF-like family receptor 1
**P01876**	IGHA1	Immunoglobulin heavy constant alpha 1
**P01877**	IGHA2	Immunoglobulin heavy constant alpha 2
**P01857;P0DOX5**	IGHG1;	Immunoglobulin heavy constant gamma 1;Immunoglobulin gamma-1 heavy chain
**P01860**	IGHG3	Immunoglobulin heavy constant gamma 3
**P01834**	IGKC	Immunoglobulin kappa constant
**A0A0C4DH25**	IGKV3D-20	Immunoglobulin kappa variable 3D-20
**P0DOY2**	IGLC2	Immunoglobulin lambda constant 2
**B9A064;P0DOX8**	IGLL5;	Immunoglobulin lambda-like polypeptide 5;Immunoglobulin lambda-1 light chain
**O95998**	IL18BP	Interleukin-18-binding protein
**P14778**	IL1R1	Interleukin-1 receptor type 1
**Q9NPH3**	IL1RAP	Interleukin-1 receptor accessory protein
**P01589**	IL2RA	Interleukin-2 receptor subunit alpha
**Q14624**	ITIH4	Inter-alpha-trypsin inhibitor heavy chain H4
**Q92876**	KLK6	Kallikrein-6
**P01042**	KNG1	Kininogen-1
**P04264**	KRT1	Keratin, type II cytoskeletal 1
**P13646**	KRT13	Keratin, type I cytoskeletal 13
**P02533;P08779**	KRT14;KRT16	Keratin, type I cytoskeletal 14;Keratin, type I cytoskeletal 16
**P13647**	KRT5	Keratin, type II cytoskeletal 5
**P32004**	L1CAM	Neural cell adhesion molecule L1

**Q16787**	LAMA3	Laminin subunit alpha-3
**O15230**	LAMA5	Laminin subunit alpha-5
**P11047**	LAMC1	Laminin subunit gamma-1
**P80188**	LCN2	Neutrophil gelatinase-associated lipocalin
**P08519**	LPA	Apolipoprotein(a)
**Q8N2S1**	LTBP4	Latent-transforming growth factor beta-binding protein 4
**P02788**	LTF	Lactotransferrin
**Q8NI32**	LYPD6B	Ly6/PLAUR domain-containing protein 6B
**Q9Y5Y7**	LYVE1	Lymphatic vessel endothelial hyaluronic acid receptor 1
**P43121**	MCAM	Cell surface glycoprotein MUC18
**Q96KG7**	MEGF10	Multiple epidermal growth factor-like domains protein 10
**Q7Z7M0**	MEGF8	Multiple epidermal growth factor-like domains protein 8
**P26927**	MST1	Hepatocyte growth factor-like protein
**P98088**	MUC5AC	Mucin-5AC
**Q9HC84**	MUC5B	Mucin-5B
**Q6W4X9**	MUC6	Mucin-6
**Q7Z3B1**	NEGR1	Neuronal growth regulator 1
**P61916**	NPC2	NPC intracellular cholesterol transporter 2
**Q9P121**	NTM	Neurotrimin
**P78380**	OLR1	Oxidized low-density lipoprotein receptor 1
**P02763**	ORM1	Alpha-1-acid glycoprotein 1
**Q8IYS5**	OSCAR	Osteoclast-associated immunoglobulin-like receptor
**Q15113**	PCOLCE	Procollagen C-endopeptidase enhancer 1
**Q5VY43**	PEAR1	Platelet endothelial aggregation receptor 1
**O75594**	PGLYRP1	Peptidoglycan recognition protein 1
**P01833**	PIGR	Polymeric immunoglobulin receptor
**Q03405**	PLAUR	Urokinase plasminogen activator surface receptor
**P00747**	PLG	Plasminogen
**P13727**	PRG2	Bone marrow proteoglycan
**Q9UKY0**	PRND	Prion-like protein doppel
**P04156**	PRNP	Major prion protein
**Q9UNN8**	PROCR	Endothelial protein C receptor
**P07602**	PSAP	Prosaposin
**P11464**	PSG1	Pregnancy-specific beta-1-glycoprotein 1
**Q9UQ72**	PSG11	Pregnancy-specific beta-1-glycoprotein 11
**Q13046**	PSG7	Pregnancy-specific beta-1-glycoprotein 7
**P41222**	PTGDS	Prostaglandin-H2 D-isomerase
**Q13308**	PTK7	Inactive tyrosine-protein kinase 7
**P15151**	PVR	Poliovirus receptor
**P11217**	PYGM	Glycogen phosphorylase, muscle form
**P02753**	RBP4	Retinol-binding protein 4
**O95980**	RECK	Reversion-inducing cysteine-rich protein with Kazal motifs

**P05451**	REG1A	Lithostathine-1-alpha
**P07998**	RNASE1	Ribonuclease pancreatic
**P10153**	RNASE2	Non-secretory ribonuclease
**O00584**	RNASET2	Ribonuclease T2
**Q8NC42**	RNF149	E3 ubiquitin-protein ligase RNF149
**Q8WZ75**	ROBO4	Roundabout homolog 4
**Q01974**	ROR2	Tyrosine-protein kinase transmembrane receptor ROR2
**P06703**	S100A6	Protein S100-A6
**P31151**	S100A7	Protein S100-A7
**Q96GP6**	SCARF2	Scavenger receptor class F member 2
**Q9NQ36**	SCUBE2	Signal peptide, CUB and EGF-like domain-containing protein 2
**Q8WVN6**	SECTM1	Secreted and transmembrane protein 1
**P14151**	SELL	L-selectin
**P04279**	SEMG1	Semenogelin-1
**P05154**	SERPINA5	Plasma serine protease inhibitor
**Q6FHJ7**	SFRP4	Secreted frizzled-related protein 4
**A6NL88**	SHISA7	Protein shisa-7
**P78324**	SIRPA	Tyrosine-protein phosphatase non-receptor type substrate 1
**Q13291**	SLAMF1	Signaling lymphocytic activation molecule
**Q99835**	SMO	Protein smoothened
**Q14515**	SPARCL1	SPARC-like protein 1
**Q9NQ38**	SPINK5	Serine protease inhibitor Kazal-type 5
**O43278**	SPINT1	Kunitz-type protease inhibitor 1
**O43291**	SPINT2	Kunitz-type protease inhibitor 2
**P22528;P35321**	SPRR1B;SPRR1A	Cornifin-B;Cornifin-A
**Q9UBC9**	SPRR3	Small proline-rich protein 3
**P52823**	STC1	Stanniocalcin-1
**P09758**	TACSTD2	Tumor-associated calcium signal transducer 2
**P02787**	TF	Serotransferrin
**O43493**	TGOLN2	Trans-Golgi network integral membrane protein 2
**P07204**	THBD	Thrombomodulin
**P07996**	THBS1	Thrombospondin-1
**P04216**	THY1	Thy-1 membrane glycoprotein
**O60235**	TMPRSS11D	Transmembrane protease serine 11D
**O14798**	TNFRSF10C	Tumor necrosis factor receptor superfamily member 10C
**Q9Y6Q6**	TNFRSF11A	Tumor necrosis factor receptor superfamily member 11A
**Q92956**	TNFRSF14	Tumor necrosis factor receptor superfamily member 14
**P19438**	TNFRSF1A	Tumor necrosis factor receptor superfamily member 1A
**P20333**	TNFRSF1B	Tumor necrosis factor receptor superfamily member 1B
**O75509**	TNFRSF21	Tumor necrosis factor receptor superfamily member 21
**P43489**	TNFRSF4	Tumor necrosis factor receptor superfamily member 4

**P22105**	TNXB	Tenascin-X
**Q9GZX9**	TWSG1	Twisted gastrulation protein homolog 1
**P07911**	UMOD	Uromodulin
**Q6EMK4**	VASN	Vasorin
**P19320**	VCAM1	Vascular cell adhesion protein 1
**Q9H7M9**	VSIR	V-type immunoglobulin domain-containing suppressor of T-cell activation
